# Process evaluation of E-learning in continuing medical education: evidence from the China-Gates Foundation Tuberculosis Control Program

**DOI:** 10.1186/s40249-021-00810-x

**Published:** 2021-03-10

**Authors:** Zi-Yue Wang, Li-Jie Zhang, Yu-Hong Liu, Wei-Xi Jiang, Sheng-Lan Tang, Xiao-Yun Liu

**Affiliations:** 1 China Centre for Health Development Studies, Peking University, Beijing, 100191 China; 2 Beijing Chest Hospital, Capital Medical University, No. 97 Ma Chang, Tongzhou District, Beijing, 101149 China; 3 Clinical Centre on Tuberculosis, Chinese Centre for Disease Control and Prevention, No. 97 Ma Chang, Tongzhou District, Beijing, 101149 China; 4 Global Health Research Centre, Duke Kunshan University, No. 8 Duke Avenue, Kunshan, 215316 Jiangsu China

**Keywords:** Continuing medical education, Training, Tuberculosis, E-learning, Process evaluation

## Abstract

**Background:**

E-learning is a growing phenomenon which provides a unique opportunity to address the challenges in continuing medical education (CME). The China-Gates Foundation Tuberculosis (TB) Control Program implemented online training for TB health workers in three provinces of China. We aim to evaluate the implementation of E-learning CME programs, analyse the barriers and facilitators during the implementation process, and to provide policy recommendations.

**Methods:**

Routine monitoring data were collected through the project office from December 2017 to June 2019. In-depth interviews, focus group discussion with project management personnel, teachers, and trainees (*n* = 78), and staff survey (baseline *n* = 555, final *n* = 757) were conducted in selected pilot areas at the provincial, municipal, and county/district levels in the three project provinces (Zhejiang, Jilin, and Ningxia). Descriptive analysis of quantitative data summarized the participation, registration, and certification rates for training activities. Thematic approach was used for qualitative data analysis.

**Results:**

By the end of June 2019, the national and provincial remote training platforms had organized 98 synchronous learning activities, with an average of 173.2 people [standard deviation (*SD*) = 49.8] per online training session, 163.3 people (*SD* = 41.2) per online case discussion. In the pilot area, 64.5% of TB health workforce registered the asynchronous learning platform, and 50.1% obtained their professional certifications. Participants agreed that E-learning CME was more economical, has better content as well as more flexible work schedules. However, the project still faced challenges in terms of unmet learning needs, disorganized governance, insufficient hardware and software, unsupported environment, and lack of incentive mechanisms.

**Conclusions:**

Our results suggested that it’s feasible to conduct large scale E-learning CME activities in the three project provinces of China. Training content and format are key facilitators of the program implementation, while the matching of training supply and demand, organizational coordination, internet technology, motivations, and sustainability are key barriers.

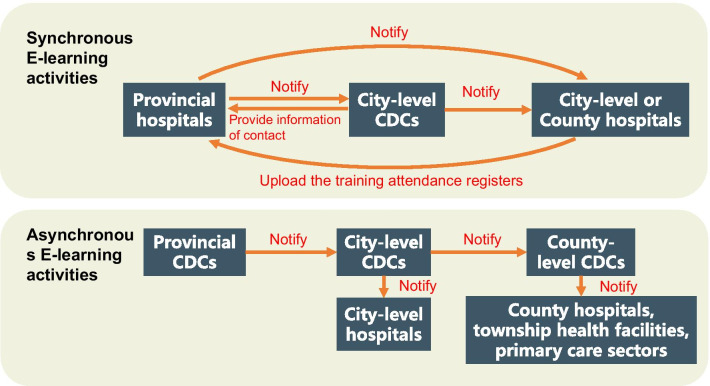

## Background

E-learning is a broad concept that involves the provision of educational programmes through electronic systems [1]. Since the 1990s, with the great progress of information technology, the E-learning market has been quickly growing around the world. In 2002, the International Data Corporation report estimated the world E-learning market was only USD 6.6 billion [2]. However, in 2018, the market size has grown to USD 190 billion and is projected to flourish into USD 423 billion by 2023 [3]. In China, the number of online education users in 2018 reached 144 million, and they downloaded online education applications (apps) more than 276 million times, which was an increase in both by a yearly rate of 20% [4]. Existing evidence indicates that E-learning could reduce cost [5, 6], improve the access to education [7] as well as provide more flexibilities for students who have work and family commitments [8, 9]. In this way, E-learning is considered to have a unique advantage in the implementation of continuing education. However, a huge volume of research on the effects did not provide information on whether effective interventions (such as E-learning) would be reproduced in specific context [10, 11]. There has been little research on E-learning implementation in continuing medical education (CME) programs, especially in low- and middle-income settings [12–14].

In the context of China, increasing demands for tuberculosis (TB) CME programs have taken place since the significant reform of China’s TB control system [15–17]. Under the new TB service delivery model, a large number of tuberculosis-related services have been transferred from the Centres for Diseases Prevention and Control (CDCs) to the designated hospitals, which highlighted a great shortage of the qualified TB health workforce [18]. At the same time, with the fast technology development in TB diagnosis, treatment, and prevention, the knowledge and skills of the existing personnel also need to be updated. These two factors have created a huge demand for CME among China’s TB control institutions. E-learning tools are increasingly available around the world. For example, The International Union Against Tuberculosis and Lung Disease (The Union) and Médecins Sans Frontières (MSF) have developed the Structured Operational Research Training Initiative (SORT IT) course, which has shown an encouraging and sustained research capacity improvement among participants [19, 20]. There is little evidence on how these E-learning tools are applied in different settings.

Since 2012, China CDC and Bill & Melinda Gates Foundation are planning to introduce and expand the new comprehensive model of TB control in China. To achieve this goal, two phases of program focused on multidrug resistant tuberculosis (MDR-TB) control, and accessibility and affordability for TB care have been implemented in 2009–2012 and 2012–2015 [21]. This project, started in 2017, named the China-Gates Foundation TB Control Program Phase 3, covered multiple complex interventions including E-learning CME for TB health personnel. We used both qualitative and quantitative approaches, to assess the participation to the E-learning CME program, to identify barriers and facilitators to implementing E-learning CME, and to provide related policy recommendations.

## Methods

### Intervention design

The E-learning project was implemented from May 2016 to June 2019 among three project provinces (Zhejiang represents the most developed eastern area in China, while Jilin is from less developed central area and Ningxia represents the least developed western area). In each province, the China-Gates program management office selected two cities as pilot areas for the E-learning project according to their level of socioeconomic development and TB health service capacity [for example, number of TB health workers, gross domestic product (GDP) per capita, level of network hardware, etc.].

Two key interventions were designed in the E-learning project of the China-Gates Foundation TB Control Program Phase 3 [22]. First, the national and provincial remote training and medical consultation mechanism was established using the "National TB Telemedicine Consultation and Training Platform", a live streaming platform developed by Clinical Centre on Tuberculosis in China CDC. This platform focused on the training of advanced diagnosis and treatment of complex clinical conditions for TB clinical staff at the county level and above (hereinafter referred to as "synchronous E-learning"). The average duration of each lecture of the synchronous E-learning is 1–1.5 h. Second, an online training and qualification system (China TB prevention Online Training Website) was developed for all TB health workers, including clinical doctors, public health physicians, and primary care medical staff. The system focused on basic knowledge and theory of TB control, with pre-recorded video tutorials. Participants can watch the video in their convenient time (hereinafter referred to as "asynchronous E-learning"). Each module of the asynchronous E-learning consists of a 25–30 min video and 5–10 questions.

The development process of these E-learning tools was led by the Clinical Centre on Tuberculosis of China CDC, which is China’s national centre for TB clinical treatment and prevention. For the synchronous E-learning, national and international experts on specific areas of TB diagnosis and treatment were selected and invited to give live lectures. For the asynchronous E-learning, a national expert group on TB control was organized to develop teaching materials based on an updated national TM control guideline. The whole process of intervention design and implementation took more than three years. The China-Gates Program has set up an advisory committee to review and evaluate the training contents.

### Study design: mixed methods of qualitative and quantitative study

This study was a qualitative study with a quantitative supplementary data. Our study results were reported according to the Consolidated criteria for Reporting Qualitative research (COREQ) reporting guidelines [23]. Site selecting criteria for intervention and sampling methods are reported in detail elsewhere [18]. Briefly speaking, the evaluation program conducted three waves of field trips (baseline, process, and final evaluation) in 2017, 2018 and 2019, respectively. We selected two cities in each of three provinces (Zhejiang, Jilin, and Ningxia) as study sites according to their level of socioeconomic development (for example, GDP per capita, types of TB health service delivery model, etc.).

Three different types of data were used in our study. First, qualitative data (key informant interviews and focused group discussions) were collected to explore the barriers and facilitators of the implementation of project. Key informant interviews (30–45 min) were conducted with project management personnel at national and provincial levels, teachers who attended training sessions, and primary care sectors. In each designated hospital and CDC, 45–60-min focused group discussions (FGDs) were conducted with 6–8 TB-related doctors, 2–3 public health physicians, or 1–3 primary care workers. The interview guide included issues on barriers and facilitators to the TB health workers’ engagement in the E-learning, the subjective effectiveness of E-learning, and the feedback in the synchronous/asynchronous E-learning from the trainers and trainees, especially compared with the traditional face to face training. The participants were recruited by coordinators from provincial and county level CDC with a purposive sampling method [24]. The interviews and FGDs were conducted in a quiet and private meeting room or office room without any other irrelevant person. A senior researcher conducted the interviews and FGDs as the interviewer or facilitator, with a junior researcher as an observer and notetaker. All interviews were recorded the after the consent form were signed. In total, we interviewed 78 participants with 17 key informant interviews and 14 FGDs in July and August 2018 (Table 1).

**Table 1:** Sample size for key informant interviews and focused group discussions

	Project management personnel	Trainers	Doctors	Public health physicians	Primary care workers
Key informant interviews (number of participants, total *n* = 17)					
National level: China CDC	NA	2	NA	NA	NA
Ningxia: Yinchuan (Capital)	1	NA	NA	1	NA
Jilin: Changchun (Capital)	2	3	NA	1	NA
Zhejiang: Hangzhou (Capital)	3	1	NA	1	NA
Zhuji (County-level)	NA	NA	1	NA	1
FGDs (number of groups, number of participants in bracket, total *n* = 14)					
National level: China CDC	1 (4)	NA	NA	NA	NA
Ningxia: Yinchuan (Capital)	NA	NA	1 (6)	NA	NA
Zhongwei (City-level)	NA	NA	1 (11)		NA
Zhongning (County-level)	NA	NA	1 (4)		1 (2)
Jilin: Changchun (Capital)	NA	NA	1 (6)	NA	NA
Jilin (City-level)	NA	NA	1 (4)	1 (3)	NA
Shulan (County-level)	NA	NA	1 (2)	1 (2)	NA
Zhejiang: Hangzhou (Capital)	NA	NA	1 (5)	NA	NA
Shaoxing (City-level)	NA	NA	1 (5)	1 (5)	NA
Zhuji (County-level)	NA	NA	NA	1 (2)	NA

*CDC* Centres for Disease Control and Prevention, *NA* not available

In addition to qualitative data, the routinely collected data from the project management office monitored all the TB institutions in the polit area from January 2017 to June 2019, which provided quantitative data for the program implementation and participation. We also performed staff questionnaires in TB health workers from three provinces during the baseline and final evaluation. In the baseline questionnaire, we collected quantitative data on participants’ demographic variables, participation and feedback of their existing CME activities (face-to-face), training need, and a quiz on TB knowledge. In the final questionnaire, we further added several questions on their participation and feedback of the synchronous and/or asynchronous online learning activities. In total, 555 TB-related health workers completed the baseline questionnaire and 757 completed the final questionnaire.

### Data analysis

For both quantitative and qualitative data, analysis was conducted for two dimensions. The first dimension was the implementation of synchronous/asynchronous E-learning (descriptive statistical analyses on participation rate of health facilities and individual health workers in the synchronous training sessions, the registration rates/certification rates for asynchronous training activities). The second dimension was the barriers and facilitators to implement the E-learning program. We transcribed the qualitative data and used a hybrid approach in thematic analysis [25]. The analytical framework was developed based on the topic guides and emerging issues from the interviews and FGDs. The quantitative data were analysed using Stata 14.0 (StataCorp, College Station, TX, USA) and the qualitative data were analysed in MAXQDA 2018 (VERBI GmbH, Berlin, German).

## Results

### Implementation of E-learning

#### Implementation of synchronous E-learning

By the end of June 2019, the national-level and three provincial training centres had conducted 98 synchronous learning activities, including 47 national-level activities (32 remote training activities and 15 case discussions). The three provincial platforms organized 51 events. Jilin had organized more activities (20) than Ningxia (13) and Zhejiang (12) (Table 2).

**Table 2:** Implementation of synchronous E-learning activities through the China-Gates Foundation Tuberculosis Control Program

Province	Times of activities			
	Remote training	Case discussions	Telemedicine consultation	Subtotal
Ningxia	13	2	2	17
Jilin	20	0	0	20
Zhejiang	12	2	0	14
National Centre	32	15	0	47
Total	77	19	2	98

Note: Data source: National TB Telemedicine Consultation and Training Platform (Dec 2017‒June 2019)

Thirty-nine TB institutions in the pilot area have access to the remote synchronous learning platform (14 in Jilin, 9 in Ningxia, and 16 in Zhejiang). On average, 23.5 TB institutions and 173.2 TB health workers accessed the national-level platform for each synchronous E-learning activity, while an average of 22.5 units and 163.3 TB health workers participated in each case discussion. Jilin had the highest participation rate followed by Ningxia. The participation in Zhejiang was relatively low (Table 3).

**Table 3:** Participation of synchronous E-learning in three provinces

Province	Average number of institutions online			Average number of students online		
	Remote training (%)	Case discussion (%)	All activities (%)	Remote training (SD)	Case discussion (SD)	All activities (SD)
Ningxia	6.1/9 (67.7)	5.3/9 (58.9)	5.8/9 (66.7)	42.8 (21.1)	36.8 (13.2)	40.9 (18.9)
Jilin	10.1/14 (72.1)	9.7/14 (69.3)	10.0/14 (71.4)	106.9 (39.4)	106.1 (34.6)	106.7 (38.0)
Zhejiang	7.1/16 (44.5)	6.8/16 (42.5)	6.9/16 (44.1)	23.5 (10.8)	20.4 (7.7)	22.5 (9.8)
Total	23.3/39 (59.7)	21.8/39 (55.9)	22.8/39 (58.5)	173.2 (49.8)	163.3 (41.2)	170.1 (47.4)

Note: Data source: National TB Telemedicine Consultation and Training Platform (Dec 2017‒June 2019)

The numerators were the number of institutions which participated in the synchronous E-learning activities and the denominators were the total number of TB institutions in polit areas

*SD* standard deviation

#### Implementation of asynchronous E-learning

By the end of June 2019, there were 23 online asynchronous training modules uploaded to the training system (7 for public health personnel, 8 for doctors, 4 for primary care workers, and 4 optional courses). Nine hundred and six (66.9%) TB health workers from the pilot area in three provinces registered for the system, and 681 (50.1%) TB health workers have obtained the qualification certificates. Zhejiang has the highest level of participation, followed by Jilin and Ningxia (Table 4).

**Table 4:** Participation of asynchronous E-learning activities in three provinces (%)

Province	Indicators	Doctors	Public health physicians	Primary care workers	Total
Ningxia	Registered the system	56/162 (34.6)	22/48 (45.8)	26/91 (28.6)	104/301 (34.6)
	Obtained the qualification certificates	23/162 (14.2)	11/48 (22.9)	13/91 (14.3)	47/301 (15.6)
Jilin	Registered the system	190/239 (79.5)	51/91 (56.0)	63/104 (60.6)	304/434 (70.0)
	Obtained the qualification certificates	123/239 (51.5)	41/91 (45.1)	51/104 (49.0)	215/434 (49.5)
Ningxia	Registered the system	60/85 (70.6)	114/151 (75.5)	324/384 (84.4)	498/620 (80.3)
	Obtained the qualification certificates	33/85 (38.8)	91/151 (60.3)	295/384 (76.8)	419/620 (67.6)
Total	Registered the system	306/486 (63.0)	187/290 (64.5)	413/579 (71.3)	906/1355 (66.9)
	Obtained the qualification certificates	179/486 (36.8)	143/290 (49.3)	359/579 (62.0)	681/1355 (50.3)

Note: Data source: China TB prevention Online Training Website (Dec 2017‒June 2019)

**Table 5:** Barriers and facilitators to implement synchronous and asynchronous E-learning activities

Domain	Themes	Related E-learning format	Description	Predefined themes
Barriers	Matching the supply and demand	Asynchronous and synchronous	Some TB health workers complained that the training materials in synchronous sessions were too difficult to learn, while some sessions were not related to their daily practices	No
	Organizational coordination	Asynchronous and synchronous	Leaders paid relatively little attention to the capacity-building subprojects, so they could not mobilize adequate resources they needed	Yes
		Synchronous	No coordinating mechanism for TB training: the CDCs and TB designated hospitals worked separately	No
	Internet technology	Synchronous	1. Professional remote equipment with a special remote centre was only available in a few provincial hospitals. Most institutions only have limited space for synchronous training. 2. The synchronous learning sessions still required all participants to join the virtual classroom at the same time, which often conflicted with their schedule	Yes
		Asynchronous and synchronous	1. Technical issues: slow processing speed of E-learning platform, network jams, system errors, fuzzy sound and blurry videos. 2. Barriers in teacher-student communications	Yes
	Motivations	Asynchronous and synchronous	(1) The lack of motivation among TB health workers due to the incentive mechanism, both extrinsic (low income) and intrinsic (lack of work motivation). (2) The incentive mechanism caused a vicious circle: low salary could only attract people with limited learning capacities and digital literacy, which further hinder them learning. (3) Still rely on external incentives such as continuing medical education credits or rules, but neither of them works very well	Yes
	Sustainability	Asynchronous and synchronous	The capacity to mobilize resources of the training organizers was very limited, which make it difficult to continue the training after the program. In addition, the platform could not charge to sustain itself due to the existing rules	Yes
Facilitators	Training format	Asynchronous and synchronous	E-learning have expanded access to high-quality continuing medical educational resources, timesaving, and reduced its costs	Yes
	Training content	Synchronous	Provincial and city-level TB health workers preferred the knowledge about disciplinary frontiers in synchronous E-learning activities	Yes
		Asynchronous	County-level TB health workers favoured sections about routine clinical practice in asynchronous E-learning activities	Yes

Note: Data source: Key informant interviews and FGDs

*TB* tuberculosis, *CDC* Centres for Disease Control and Prevention

**Fig. 1:**
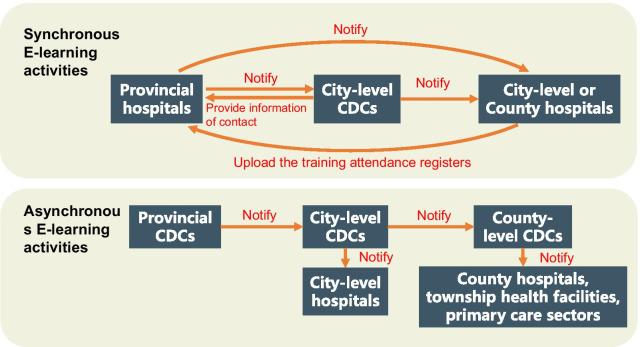
Organizational structure of capacity building in Zhejiang. *CDC* Centre for Disease Control and Prevention

**Fig. 2:**
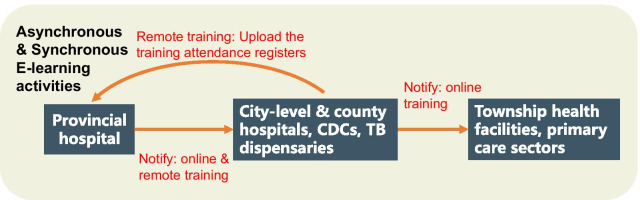
Organizational structure of capacity building in Jilin and Ningxia. *CDC* Centre for Disease Control and Prevention, *TB* tuberculosis

Key informants reported that different provinces had different patterns of training organization. In Zhejiang Province, asynchronous learning activities were mainly organized by the CDCs with the "province-city-county" three-tier administrative management mode, while synchronous learning activities were TB designated hospitals’ responsibility. The organization of synchronous learning activities was led by provincial designated hospital. Since there is no vertical administrative relationship between the designated hospitals, the provincial hospitals could not effectively coordinate the training activities. Instead, they had to rely on local CDCs as intermediary partners to send training reminders and other messages (Fig. 1). In Ningxia and Jilin, all institutions were notified of synchronous/asynchronous learning activities by provincial hospitals (including CDCs, hospitals, and TB dispensaries), while trainees at the township level were notified by county-level CDCs (Fig. 2).

### Facilitators of E-learning CME

#### Facilitators of the training format

In the interviews, most TB health workers agreed that the E-learning model is economical and timesaving compared to traditional face to face training. In the past, only a few face-to-face training opportunities were available for TB health workers, especially for those from lower-level institutions. The TB institutions could only send one or two doctors to attend the training and forward the training contents for those who were not able to attend. They often used meetings as a substitute for on-job training. E-learning have expanded access to high-quality continuing medical educational resources, and reduced its costs.

*Compared with the traditional training model, the online and remote training are timesaving and more efficient. Traditional methods are not able to invite people come over frequently, and most hospitals can't afford the human and financial costs. (Provincial TB health professional FGD in Zhejiang)*

#### Facilitators of training content

In general, the training contents of the China-Gates Program were regarded as high-quality by the TB health workers. The trainers of E-learning were well-known experts in China. The lower-level health workers could receive training and technological guidance from the national experts, which was unlikely in traditional face-to-face training. Different health workers have different preferences: provincial and city-level TB health workers preferred the frontiers and advances in synchronous E-learning activities, while county-level TB health workers favoured sections about routine clinical practice in asynchronous E-learning activities.

*It's really nice to stay at home and have direct access to teachers from Beijing or across the country, teaching us the basic knowledge or cutting-edge ideas. It’s brilliant. (Provincial TB health professional FGD in Jilin)* (Table 5)

### Barriers in the implementation of E-learning

#### Barriers in matching the supply and demand of training

Although China-Gates Program provided TB staff with high-quality training contents, they were not evenly spread out among different types of TB health workers. In general, more courses were available to the clinical doctors: the total training loads including synchronous session once a week (or twice monthly) and nine modules of asynchronous sessions. However, the training loads for public health physicians (seven modules in total) and primary care workers (four modules in total) was relatively small.

The uneven supply and demand was another key concern. Staff survey showed that clinical doctors thought they need more training in the clinical diagnosis and treatment of TB (83.5% for multidrug-resistant TB, MDR-TB, 78.4% for TB radiology and 70.1% for common TB), which perfectly matched with their training supplies (Table 6). Public health physicians expected more training in TB infection control (76.2%), planning (73.4%), and surveillance (63.1%), they mostly received training in planning (91.8%), TB management (89.8%) and treatment of common TB (75.5%). Similar results were observed in FGDs. Some TB health workers complained that the training materials were too difficult to follow and some sessions were not related to their daily practices.

*The content of remote (synchronous) training is too deep to be used in clinical practice, which is beyond our competence, especially for county-level personnel. (County-level TB health professional FGD in Jilin)*

**Table 6:** Supply and demand of training among tuberculosis (TB) health workers. Data source: TB health worker survey (first quarter in 2017 and third quarter in 2019)

Type of TB health workers	Training demand		Training supply		Matching
	Sample size	Top three most mentioned (%)	Sample size	Top three most mentioned (%)	
Doctors	97	Treatment for MDR-TB (83.5)	136	Treatment for common TB (90.4)	Yes
		TB radiology (78.4)		TB radiology (85.3)	Yes
		Treatment for common TB (70.1)		Treatment for common TB (85.3)	Yes
Public health physicians	65	TB planning (73.4%)	49	TB planning (91.8)	Yes
		TB infection control (76.2)		TB management (89.8)	No
		TB surveillance (63.1%)		Treatment of common TB (75.5%)	No
Primary care workers	267	Treatment for common TB (79.8)	164	Treatment for common TB (90.2)	Yes
		Treatment for MDR-TB (70.4)		TB case detection (72.6)	No
		TB management (66.7%)		TB management (83.5)	Yes

*MDR-TB* Multidrug resistant tuberculosis

### Barriers in organizational coordination

The first challenge in organizational coordination was that the leaders did not pay enough attention to training. Interviews with project management personnel in Zhejiang and Ningxia provinces revealed that they could not mobilize adequate resources due to the low relative priority of training among leaders’ goals. On the contrary, the areas with higher levels of leaders’ attention such as Jilin reported higher level of training participation.

*In our case, to be honest, we need to take care of everything. We are extremely busy…… For example, online training (asynchronous E-learning) is only a very small part of the China-Gates program, so we can't put lots of resources on it…… We can’t be anywhere; this is our situation. (Key informant interview of CDC leader in Zhejiang)*

The second challenge in organizational coordination was how the CDCs and TB designated hospitals collaborate on organizing training, providing feedback, and solving problems. As we mentioned in Fig. 1, there was no effective coordination mechanism between CDCs and designated hospitals for TB CME. It hindered further expansion of the high-quality learning resources.

### Barriers in internet technology

E-learning CME placed higher demands on hardware and software. According to the survey, there were three kinds of hardware equipment for synchronous E-learning: a few provincial hospitals had well-equipped special training centre; In most city-level hospitals, physicians used desktop computers and a projector to access to the E-learning platform as a group; However, in the most county hospitals, physicians could only use their personal computers in the office. In addition, technical issues arose when TB health workers received training, such as the slow processing speed of E-learning platform, network jams, system errors in learning progress, fuzzy sound and blurry videos.

*Because we're not using a special-purpose device, sometimes the internet connection is not working. It happens sometimes that we could not join the (synchronous E-learning) classroom. (County-level TB health professional FGD in Jilin)*

Another challenge came from the technology itself. Firstly, most medical staff feel that E-learning should not be a substitute for face-to-face training because of the barriers in teacher-student communication. Secondly, although they didn’t have to leave their work space for E-learning activities, the synchronous learning sessions still required all participants to join the virtual classroom at the same time. Many medical staff reflected that the training often conflicted with their work schedule, and they could not leave their office to participate in training during the working days.

### Barriers in motivations

The lack of motivation was another source of barrier for training. The incentive mechanism in current TB health service delivery system in China has a sustained negative impact on TB health workers. Firstly, the medical institutions largely depend on services revenues to cover operational costs. However, according to the national TB control program, the TB departments are funded by the government to provide free medical services, they have limited capacity to generate revenues. In this way, TB health workers have lower income and lack of work motivation. Moreover, participation in training has no association with the promotion or payments, which makes medical personnel more reluctant to take part in training. Sometimes one single person in the department was asked to participate all the training, and signed up for every colleagues. Secondly, due to poor income, the TB workforce in the three provinces were generally older, with lower professional titles, and less education compared with their colleagues in other departments. Their learning capacities and digital literacy were relatively weak.

*When you are not satisfied with your revenue, you don't have the motivation to work, you just deal with everything…… our clinical physicians, especially our heads of the clinical departments, they seem to feel that, managing patients, performance, and getting more money—he feels that that's the point, training is not important. (Key informant interview of project management personnel in Ningxia)*

Since TB health personnel were more likely to be “passive learners” instead of “active learners”, the E-learning activities in many institutions still rely on external incentives such as CME credits. However, some project management personnel reflected that due to the stringent regulation of CME credits, it’s difficult for TB health workers to get credits from the China-Gates training project. They could only try other strategies such as sending repeated notifications to doctors or forcing them to participants, but neither of them works well.

### Barriers in project sustainability

Many stakeholders expressed their concerns about the sustainability of the training. The capacity to mobilize resources of the training organizers was very limited: both national and provincial platforms are facing the tight constrains in terms of funding and human resources, which make it difficult to mobilize resources to continue the training after the program is completed. Additionally, government regulations stated that hospitals cannot charge patients for telemedicine consultations. Therefore, the platform has no existing model to raise funding to sustain itself.

## Discussion

This study has aimed to analyse the barriers and facilitators to implementing E-learning in continuing medical education in three provinces of China. It found that the overall progress of the E-learning project of the China-Gates Foundation TB Control Program has been relatively smooth. According to the pre-set plan, 98 synchronous learning activities were conducted by the national and provincial platforms. The registration rate of the asynchronous online platform for TB professionals in the pilot areas has exceeded 60%. In general, the project has achieved the goal of transferring high-quality medical educational resources to grass-roots medical institutions. However, the project still faces a range of challenges in the implementation, such as unmet training needs for public health and primary care workers, inefficient management framework, insufficient hardware and software, the unsupported environment, and the lack of an incentive mechanism. The current training model has not satisfied the needs for long-term, systematic, and timely training of TB professional.

The China-Gates Foundation TB Control Program implemented a large-scale E-learning program in TB continuing medical education. Compared to the traditional face-to-face training, this E-learning program has several advantages in content and format of training: (i) This project invited national-level famous experts to give lectures, the content of lectures was revised by several waves of the experts’ consultation with academics across the country. (ii) TB medical personnel could stay at home to receive training from state-level experts and the high-quality educational resources has transferred to the grassroots level. Although it will not replace face to face training in the future, we did see great feasibility and necessity of E-learning, especially during the COVID-19 pandemic [26]. In addition, some blended learning program for TB researchers from low- and middle-income countries (LMICs), like the SORT IT course, has shown an encouraging and sustained research capacity improvement among participants [19, 20, 27, 28]. With the improved internet access in the future, it’s possible that E-learning will be components of the routine practices in CME.

Based on previous studies, E-learning has two contradictory effects: first, compared with traditional face-to-face learning, students in E-learning environment were more likely be inferred by online environment and lack of personal discipline, which has the negative effect on students’ performance. This phenomenon was well studied by many researches in high-level education where the students could acquire same teaching resources online and offline [5, 29–33]. Second, E-learning could expand access to education which is good for people from the underrepresented group. Only few studies realized the importance of accessibility improvement by E-learning [7], evidence shown that some teaching physicians from LMICs favoured the E-learning's unprecedented value in knowledge transmission and accessibility [34]. Since the high-quality tutorial was one of the main facilitators for China-Gates capacity building program, we argue that the second effect played a more important role in our study—many health workers could not take in-person classes from national experts. In further analyses, we did find some evidence that the program improved the knowledge of TB among primary care providers, probably due to the second phenomenon we mentioned (Wang et al. 2021, under review).

Our results highlight the lack of internal training incentives and low self-efficacy among TB personnel as one of the most important barriers. This phenomenon was caused by the policy environment and organizational culture, especially the incentive mechanism. In most study areas, the implementation of training programs still relied on external incentives, such as continuing medical education credits. Extrinsic motivations (continued medical education credit, attendance requirement) are important in the short terms, but the long-term success of training project depend on whether we can engage health workers as active participants in their learning. How to establish and strengthen the virtuous cycle of intrinsic motivation is worth further studying.

Different types of training have their own advantages, which are influenced by the type of course, the quality of the content, the communication between teachers and students, and the flexibility of time and space. Existing evidence showed that compared with face-to-face learning, E-learning has stronger negative effect for applied professional courses (e.g., business, law, and nursing), but little impact on theoretical courses such as philosophy and psychology [35]. It suggested that face-to-face training is more suitable for operational courses, which not only allows students to have hands-on opportunities, but also facilitates communication between teachers and students. However, for theoretical courses, quality educational resources are scarce, and medical personnel have to travel for training while they have work commitments. In this case, the advantages of E-learning became apparent. Therefore, E-learning and traditional face-to-face learning are complementary. The implementations of other pedagogies with a combination of face-to-face learning and E-learning in CMEs, such as blended learning [36], is a question for future studies.

Based on these findings, we proposed five policy recommendations. The first is to improve the organizational structure of training. All TB institutions should pay more attention to training and to create a learning culture of teams. Second, to promote multi-sectoral collaboration. In the context of “the trinity model” and as institutions with hierarchical administration, CDCs should assist the designated hospitals in this process. More management and coordination functions should be transferred from CDCs to designated hospitals, enhancing their abilities and willingness to train and serve hospitals at lower levels. Third, to strengthen the supervision and establishing effective incentive mechanisms. We suggest to add continuing medical education credits at the design stage, allowing primary care workers to obtain national credits from the basic courses, and to decentralize the authority of credits in advanced courses to the provincial and municipal levels. Fourth, to enhance the user experience through technical assistance. We would like to highlight several key elements: optimizing the operating speed of the training platform, adding resume breakpoint and the video replay function, setting up a forum to facilitate teacher-student communication and interaction, developing mobile apps, and conducting a survey on user behaviours in order to simplify the user interface and operation. Finally, the fifth recommendation is to create customized and diverse learning experiences. Designing blended learning modules to combine the online and offline courses needs to be considered. Doctors can selectively attend training sessions according to their own needs and schedules.

There are two main limitations in our studies: first, this study was performed within a specific group—health workers specialising in TB prevention and control, who were likely to have less than average interest due to the policy environment, and thus, the conclusion maybe inconsistent within other groups. In addition, we did not have the baseline information for synchronous E-learning participants, so we could not calculate the participation rate of synchronous E-learning activities as we did in the analyses of the asynchronous one.

## Conclusions

It’s feasible to conduct large scale E-learning continuing medical education activities with TB health personnel in China. Training content and format seem to be key facilitators of the program implementation, while the matching the supply/demand of training, organizational coordination, internet technology, motivations, and sustainability are key barriers. Further research should consider the theory behind the sperate effects more carefully in order to set up priorities for E-learning activities in CME.

## Data Availability

The datasets used and/or analysed during the current study are available from the corresponding author on reasonable request.

## Abbreviations

apps: Applications
CDC: Centre for Disease Control and Prevention
CME: Continuing medical education
COREQ: Consolidated criteria for Reporting Qualitative research
COVID-19: Coronavirus disease 2019
FGDs: Focused group discussions
GDP: Gross domestic product
MDR-TB: Multidrug resistant tuberculosis
*SD*: Standard deviation
SORT IT: Structured Operational Research Training Initiative
TB: Tuberculosis
